# The Sexual Motivation of Male Rats as a Tool in Animal Models of Human Health Disorders

**DOI:** 10.3389/fnbeh.2019.00257

**Published:** 2019-11-26

**Authors:** Michal Bialy, Wiktor Bogacki-Rychlik, Jacek Przybylski, Tymoteusz Zera

**Affiliations:** ^1^Department of Experimental and Clinical Physiology, Laboratory of Centre for Preclinical Research, Medical University of Warsaw, Warsaw, Poland; ^2^Department of Biophysics and Human Physiology, Medical University of Warsaw, Warsaw, Poland

**Keywords:** sexual motivation, general arousal, sexual arousal, ultrasonic vocalizations, depression, anxiety, metabolic disorders, male behavior

## Abstract

Normal or dysfunctional sexual behavior seems to be an important indicator of health or disease. Many health disorders in male patients affect sexual activity by directly causing erectile dysfunction, affecting sexual motivation, or both. Clinical evidence indicates that many diseases strongly disrupt sexual motivation and sexual performance in patients with depression, addiction, diabetes mellitus and other metabolic disturbances with obesity and diet-related factors, kidney and liver failure, circadian rhythm disorders, sleep disturbances including obstructive sleep apnea syndrome, developmental and hormonal disorders, brain damages, cardiovascular diseases, and peripheral neuropathies. Preclinical studies of these conditions often require appropriate experimental paradigms, including animal models. Male sexual behavior and motivation have been intensively investigated over the last 80 years in animal rat model. Sexual motivation can be examined using such parameters as: anticipatory behavior and 50-kHz ultrasonic vocalizations reflecting the emotional state of rats, initiation of copulation, efficiency of copulation, or techniques of classical (pavlovian) and instrumental conditioning. In this review article, we analyze the behavioral parameters that describe the sexual motivation and sexual performance of male rats in the context of animal experimental models of human health disorders. Based on analysis of the parameters describing the heterogeneous and complex structure of sexual behavior in laboratory rodents, we propose an approach that is useful for delineating distinct mechanisms affecting sexual motivation and sexual performance in selected disease states and the efficacy of therapy in preclinical investigations.

## Introduction

Sexual interaction has been one of the most intensively studied appetitive behaviors over the last 80 years. Copulation differs between species, but detailed investigation of the mechanisms regulating the behavior of one species seems to be important from the perspective of comparative physiological research. Furthermore, effective sexual interactions involve activation of a sequence of behavioral patterns that depend on distinct brain structures, neural networks, and neurotransmitters. The amygdala (A), bed nucleus of stria terminalis (BNST), medial preoptic area (MPOA), and central tegmental field/subparafascicular nucleus of the thalamus constitute the core central structures. They connect with the dopaminergic mesolimbical, mesocortical, and nigrostriatal tracts, lateral and ventromedial hypothalamus, paraventricular nucleus of the hypothalamus, ventral premammilary nucleus, midbrain periaqueductal gray, nucleus paragigantocellularis of the medulla, and autonomic regions of the spinal cord and regulate sexual motivation, arousal, and copulatory performance. Detailed analysis of neural networks and neurotransmitters in the context of sexual behavior is outlined in several recent reviews (Hull and Rodríguez-Manzo, 2017; Hill and Elias, 2018; Seizert, 2018; Le Moëne and Ågmo, 2019).

With this background, analysis of the sexual activity of laboratory rodents provides a powerful experimental tool for studying the inheritable traits, endocrine factors, neurotransmitter systems, and neural networks involved in evolutionarily preserved as well as experience-dependent aspects of behavior.

In this review article, we analyze the behavioral parameters describing the sexual motivation and sexual performance of male rats in the context of health disorders in humans. Based on analysis of parameters describing the heterogeneous and complex structure of sexual behavior in laboratory rodents, we propose an approach that is useful for delineating distinct mechanisms affecting sexual motivation and sexual performance in disease states and the efficacy of therapy in preclinical investigations. Furthermore, we argue that this approach could be applied for more precise determination of specific mechanisms involved in abnormal or disturbed sexual behavior in rats that are translationally related to human health disorders. In particular, translational research in rodent models of sexual behavior has provided important insights into the pathomechanisms and pharmacotherapy of clinical conditions that are described in the Diagnostic and Statistical Manual of Mental Disorders 5th edition (DSM 5) and in the International Statistical Classification of Diseases and Related Health Problems 10th revision (ICD-10), including premature ejaculation, paraphilias, mood and anxiety disorders as well as neurological and metabolic diseases (McVary et al., 1997; Grønli et al., 2005; Giuliano and Clément, 2006; Hawley et al., 2013; Pfaus et al., 2013; Kang et al., 2014; Olayo-Lortia et al., 2014; Sanna et al., 2014; Faulkner et al., 2015; Babaei-Balderlou and Khazali, 2016; Oosting et al., 2016; Ramírez-Rodríguez et al., 2017; Hernández and Fernández-Guasti, 2018; Novati et al., 2018). In this light, we propose that in various rodent models of human disease states, sexual motivation and performance may be differently affected, which is reflected in distinct changes of specific components of male rat sexual behavior. However, this translational potential of animal models of sexual behavior for investigating human disorders should be exploited cautiously, as not all aspects of sexual behavior and health disorders are identical in rats and humans (Le Moëne and Ågmo, 2019). Here, we present an outline of male rat sexual behavior in the context of rodent models of human diseases, which should be helpful in finding appropriate experimental models for evaluation of pathomechanisms, therapeutic interventions, and alternatives to the current therapies in preclinical studies. Owing to the specificities and differences of male and female sexual motivation and behavior under physiological conditions and in health disorders (Pfaff, 2017; Hill and Elias, 2018), we did not analyze female sexual behavior in the review.

## Sexual Behavior as an Experimental Model

The sexual behavior of male rats consists of the anticipatory stage during which a male searches for a receptive female, followed by an initiation stage during which a male and a female show mutual investigation. At the end of the initiatory stage, rats begin to copulate. Female behaviors, including sex-soliciting behavior, receptivity, and occurrence of the lordosis reflex (measured as % displaying lordosis), influence the initiation stage and copulatory performance, as lordosis allows for intromission. Copulation comprises specific highly stereotypical motor patterns, including mounting, intromission, and ejaculation. Mounting is a pattern when a male lifts his forebody over the female hindquarters, clasping her flanks with his forepaws, and begins a series of rapid shallow movements of the pelvis. When the glans penis detects the vagina, a male can perform rapid erection, with a deeper intravaginal thrust, which is followed by immediate dismounting. This mounting-intromission-dismounting pattern is repeated until ejaculation is achieved. Ejaculation is marked by a long-lasting intromission (about 1–2 s), which a male rat usually achieves after a few to a dozen intromissions. Rats usually copulate for up to eight ejaculations until copulatory satiation (Larsson, 1956; Sachs and Barfield, 1976). During all these stages, rats produce a complex series of ultrasound vocalizations of various frequencies and temporal patterns (Barfield et al., 1979). Furthermore, fully expressed sexual behavior requires both sexual motivation and sexual arousal, which should be treated as two distinct phenomena (Sachs, 2000). Sexual arousal depends on the activation of brain networks within the brainstem that simultaneously control behavioral and autonomic nervous system responses during sexual interaction and is mainly manifested by penile erection, whereas sexual motivation drives and maintains subsequent stages of sexual behavior (Schober et al., 2011; Ågmo, 2011). Differences between sexual motivation and sexual arousal, or more precisely the fact that these processes are not interchangeable or equivalent, can be explained by the analysis of non-copulating male rats or asexual orientation in humans. In healthy non-copulating rats, noncontact erections are present during exposure to the receptive female in spite of the absence of an attempt to copulate, which is especially visible after medial preoptic lesions (Stefanick and Davidson, 1987; Liu et al., 1997; Portillo and Paredes, 2019). Similar dissociation between sexual arousal and sexual motivation can be seen in asexually oriented men. The level of masturbation in healthy asexual men is similar to that in heterosexual counterparts, but with no motivation for either hetero or homosexual interactions (Brotto et al., 2010; Portillo and Paredes, 2019).

**Figure 1:**
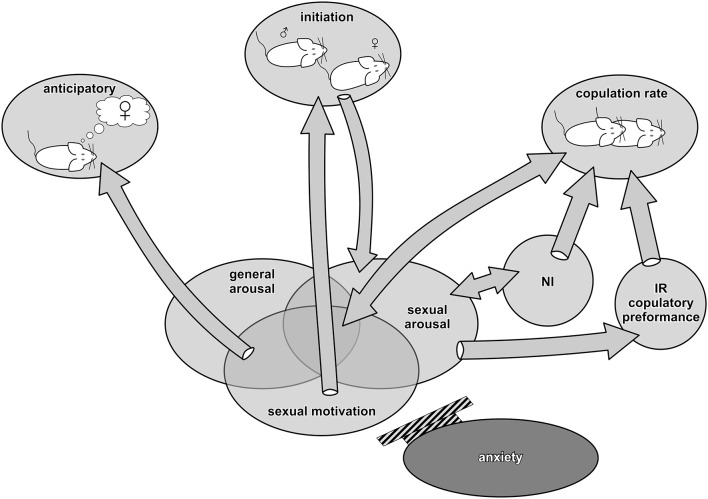
Components—factors of male sexual behavior in relation to sexual motivation, general arousal, and sexual arousal. Anticipatory behavior is related to sexual motivation and general arousal. The initiation phase is powered by sexual motivation and leads to the enhancement of sexual arousal necessary to evoke penile erection. Anxiety inhibits this phase. Copulation rate is related to the reciprocally augmented level of sexual motivation and sexual arousal as well as to general arousal. NI, number of intromissions, is reciprocally related to sexual arousal accumulation and affects the copulatory rate. IR, intromission ratio, is dependent on erectile function/dysfunction and strongly affects copulatory efficiency.

The sexual behavior of a male rat contains both inheritable arousal-activated neuronal networks and networks that are experience-dependent and modified by classical and instrumental conditioning (Pfaus et al., 2001; Hull and Rodríguez-Manzo, 2017). Based on statistical factor analysis of sexual behavior in copulatory experienced rats, five independent components of sexual behavior have been distinguished: anticipatory, initiation, rate of copulation, number of intromissions, and intromission ratio (IR; Sachs, 1978; Pfaus et al., 1990). All five components of sexual interactions are summarized in Figure 1 and discussed below from the perspectives of sexual motivation, general arousal, and sexual arousal.

### Anticipatory Behavior

In standard laboratory procedure, a male rat is usually placed in the experimental chamber for 5 min before introduction of a receptive female (Larsson, 1956). During this time, anticipatory behavior is measured by intensiveness of chamber exploration, number of rearings, or changes of levels in special bi-level apparatus (Mendelson and Gorzalka, 1987; Mendelson and Pfaus, 1989). In sexually experienced males, intensiveness of exploration with looking for cues from a female co-occurs with intensive ultrasonic vocalizations in the 50-kHz band, termed precontact vocalizations (PVs; Bialy et al., 2000). Ultrasounds emitted by rodents, in addition to olfactory cues, are a signal for identification of individuals (Holy and Guo, 2005; Asaba et al., 2014). Ultrasonic vocalizations in the 50-kHz band reflect the emotional state of rodents and are related to the activation of the nucleus accumbens (Hamed et al., 2016; Mulvihill and Brudzynski, 2018). Ultrasounds also cause rats to react by approaching a sound source (Wöhr and Schwarting, 2007; Pultorak et al., 2016). The number of PVs during the acquisition of sexual experience is related to the level of sexual experience and conditioning to odor cues and conditioning stimuli (CS) from the experimental chamber, and it depends on the rewarding value of sexual contacts (Bialy et al., 2000).

PVs and other elements of anticipatory behavior can be completely suppressed by repeated dopamine D1 receptor activation in the nucleus accumbens without significant effects on subsequent copulatory behavior (Bialy et al., 2010). Furthermore, increase in the number of PVs during the acquisition of sexual experience is inhibited by NMDA antagonists but is not related to the initiation of copulation measured by mount latency (ML; see below; Bialy et al., 2000). These observations indicate that anticipatory behavior depends on different neural networks than initiation and copulatory behaviors.

In the sexual context, penile erection is treated as an indicator of an elevated level of sexual arousal (Sachs, 2000). However, our studies indicate that penile erection is not observed during anticipatory behavior (Bialy et al., 2000, 2010). This implies that the anticipatory behavior depends on stimulation of the general arousal system and motivation to look for cues related to sexual activity rather than on the sexual arousal itself.

### Initiation Behavior

The time between exposure of a male rat to a receptive female and the first mounting determines the length of the initiation stage and is described as ML. When a female is introduced, both male and female show mutual investigation and mutually emit ultrasonic vocalizations. Odor, visual, auditory, and tactile cues enhance both the level of sexual motivation, leading to copulatory behavior, and the level of sexual arousal, making it sufficient to evoke erection and effective intromission (Hull and Rodríguez-Manzo, 2017). ML depends on sexual motivation enhanced by mutual male-female investigation. In addition to the motivational aspect measured by ML, the latency between the introduction of a female to the first intromission, termed intromission latency (IL), indicates the time required to reach a sufficiently high level of sexual arousal to induce erection (Hull and Rodríguez-Manzo, 2017). ML is prolonged in sexually naïve males and is significantly shorter in sexually experienced rats. Sexual experience and conditioning to cues from a female or experimental chamber significantly reduce both ML and IL (Larsson, 1959; Dewsbury, 1969; Bialy et al., 2000).

The initiation of copulation is strongly related to sexual motivation, and it is inhibited by an enhanced level of anxiety (Pfaus and Wilkins, 1995; Miwa et al., 2011). Activation of cAMP-response element-binding protein (CREB) in the nucleus accumbens reduces anxiety level and ML, but it has no effect on copulatory efficiency as measured by ejaculation latency (EL; Barrot et al., 2005). Similarly, acute administration of D1 receptor agonist into the nucleus accumbens significantly increases the percentage of sexually naïve males that display mounting, but without an increase in PVs or shortening of EL (Bialy et al., 2010). ML is also dramatically prolonged by lesion of the anterior cingulate cortex (Ågmo et al., 1995), suggestive of a critical role for the nucleus accumbens–anterior cingulate cortex/medial prefrontal cortex network in sexual motivation and initiation of a new behavior (Bialy et al., 2010; Sanna et al., 2017). Sexual motivation can also be described by approach behavior in the sexual incentive motivation test arena (Le Moëne and Ågmo, 2019).

### Copulatory Efficiency (Copulatory Rate Factor)

Sexual motivation during copulation controls the behavior directed towards the satisfaction of sexual drive by achieving intromissions and ejaculations. Copulatory efficiency describes the ability to satisfy the sexual drive and depends on sexual experience (Larsson, 1959; Dewsbury, 1969; Bialy et al., 2000). Simultaneously with sexual motivation, adequate sexual arousal has to be achieved and accumulated to elicit penile erection and ejaculation, which are mediated by activation of the autonomic nervous system (Giuliano and Rampin, 2004).

The most important measure of copulatory efficiency is EL, which describes the time from first intromission to ejaculation. Additionally, copulatory efficiency can be measured by inter-intromission interval (III), which provides the mean time between intromissions before each ejaculation (Sachs and Barfield, 1976; Sachs, 1978).

Acquisition of sexual experience has been shown to involve different neuronal networks in copulatory efficiency and in the initiation of copulation (Bialy et al., 2000, 2010). The key neural network that regulates copulatory rate involves connections between the amygdala, BNST, central tegmental field, and MPOA (Hull and Rodríguez-Manzo, 2017). MPOA is one of the brain structures in which acquisition of sexual experience leads to an increase in Fos expression, and higher levels of neurotransmitters, receptors, or enzymes and hormones important for regulation of sexual activity (Hull and Rodríguez-Manzo, 2017). Specifically, it was shown that acquisition of sexual experience is associated with an increase in nitric oxide with higher levels of nitric oxide synthase (Dominguez et al., 2006), glutamate and dopamine (Will et al., 2014), D1 receptor signaling (McHenry et al., 2012) and D2 receptor signaling (Nutsch et al., 2016), and oxytocin receptors (Gil et al., 2013), and an increased number of neurons containing androgen receptors (Swaney et al., 2012). Moreover, acquisition of sexual experience and improvement in copulatory efficiency was shown to involve neuronal plasticity as measured by *c-fos* expression in the parieto-occipital cortex (Bialy et al., 1992; Bialy and Kaczmarek, 1996). Additionally, dopamine and noradrenaline levels in the medial prefrontal cortex correlate with sexual experience (Sanna et al., 2017).

### Number of Intromissions

The number of intromissions indicates the level of genital stimulation required to induce ejaculation and describes the accumulation of sexual arousal. The cortico-medial part of the amygdala accumulates arousal in rats (de Jonge et al., 1992), as lesions in this region lead to a dramatic increase in number of intromissions (Harris and Sachs, 1975). Similar effects were observed after BNST lesion (Valcourt and Sachs, 1979). Strong reductions in intromission number were observed after serotonin 5HT1A receptor agonist (Snoeren et al., 2014) and D2 agonist and, less effectively, D1 agonist, but not D4 agonists (Cagiano et al., 1989; Beck et al., 2002; Sanna et al., 2015).

### Intromission Ratio

Males display intromissions and/or mounts without intromission from the initiation of copulation to ejaculation. The IR describes the proportion of intromissions to the sum of mounts and intromissions. A low value of this parameter is strongly related to erectile dysfunction, which may be due to a low level of NO synthesis (neuronal and epithelial source), peripheral neuropathy, or vascular pathology (Hull et al., 1994; Bialy et al., 1996).

Evaluation of copulatory efficiency is critically important in rat models of premature ejaculation. Short ejaculation latencies with a very low number of intromissions (1 or 2 in a copulatory series) were observed in rats treated with 5HT-1A agonist, which can be considered a model of premature ejaculation (Coolen et al., 1997). Another model of premature ejaculation is based on the fact that in sexually experienced rats, there are two extreme endophenotypes. One represents premature ejaculation, with male rats achieving rapid and frequent ejaculations, up to five ejaculations during a short 30-min session of sexual interactions. The other phenotype represents the animal model of retarded ejaculation, with sexually experienced rats achieving only intromissions without any ejaculations during such a session (Pattij et al., 2005a; Waldinger and Olivier, 2005). Such model can be useful for understanding the mechanisms and pharmacological background of premature ejaculation and the role of serotonin receptors, selective serotonin reuptake inhibitors (SSRI), and oxytocin receptors (Giuliano and Clément, 2006; Clément et al., 2007, 2012, 2013; Kang et al., 2013, 2014; Oosting et al., 2016) but only in the case when less genital stimulation is required to achieve ejaculation (fewer intromissions). However, findings from our laboratory indicated that the majority of normal male rats were capable of achieving extravaginal ejaculations when mounting a female with a closed vaginal orifice, provided the male rats received sufficient genital stimulation during at least two intromissions preceding the extravaginal ejaculation. Furthermore, this phenomenon was independent of the number of mountings and was present without any pharmacological intervention (Beck and Bialy, 2000).

In addition to the retarded ejaculation model described above, copulatory efficiency and sexual motivation are strongly affected by metabolic disorders, especially type 2 diabetes mellitus (McVary et al., 1997; Faulkner et al., 2015), depressive-like/anhedonic states (Pfaus and Phillips, 1991; Van Furth et al., 1994; Bialy et al., 2014), or high anxiety levels (Hawley et al., 2013; Sanna et al., 2014).

### Postejaculatory Behavior

The mechanisms behind the postejaculatory period are relatively poorly understood and involve numerous spinal and supraspinal structures of the central nervous system (Seizert, 2018; Le Moëne and Ågmo, 2019). In the postejaculatory period, all three processes: general arousal, sexual motivation, and sexual arousal, which control the male’s behavior are reflected in different parameters. After ejaculation, a male usually moves to one of the corners of a chamber and starts to emit a vocalization in the 22-kHz band. Most of the time, a male does not move when vocalizing. About 2 min after the first ejaculation (and significantly later after the second one), a male starts to explore the experimental chamber, even before termination of the postejaculatory vocalizations (Sachs and Bialy, 2000). This exploratory behavior reflects increasing general arousal but not sexual motivation or sexual arousal. Operant behavior shows that, at this time, male rats very often perform instrumental reactions—bar-pressing or run in a runway—but that after arriving in the compartment with a female, they evidently escape any socio-sexual contact and show a departure reaction (Beck, 1986; Beck et al., 2002). On the other hand, sexual arousal measured by penile erection occurs later than first exploratory behavior, at least during the first postejaculatory period. Such erection is visible even before the termination of postejaculatory 22-kHz vocalizations, suggesting that sexual arousal increases before a male starts to show interest in a female due to enhanced sexual motivation (Sachs and Bialy, 2000). Sexual motivation and interest in a receptive female appear after the termination of vocalizations. Furthermore, weak painful stimuli that increase sexual motivation in a non-specific way enhance a male’s interest in a female and mating, but this is present only after the end of postejaculatory vocalizations (Sachs and Barfield, 1974, 1976). These findings suggest that sexual motivation during postejaculatory ultrasonic vocalizations remains at a very low level. Therefore, after ejaculation, three parameters, latency to the first exploration, latency to the first noncontact erection, and latency to approaching a female, can be treated as measures of general arousal, sexual arousal, and sexual motivation, respectively. The initiation of enhanced sexual motivation later than of enhanced sexual arousal indicates that the postejaculatory interval is not simply the mirror state of the anticipatory and initiation phases of sexual behavior. Ultrasonic postejaculatory vocalizations, on the other hand, reflect, in our opinion, a relaxation state after ejaculation (Bialy et al., 2016). An enhanced level of general arousal and sexual arousal before the termination of postejaculatory vocalizations can be distinguished by spectral analysis of postejaculatory calls. Before the termination of vocalizations, at the time as exploration or noncontact erection take place, some frequency modulations or a shift from about the 45-kHz to the 28–23-kHz band are more often detected, and these differ from the very flat 22-kHz frequency ultrasonic vocalizations at the beginning and middle of the postejaculatory period (Bialy et al., 2019).

### Rewarding Value of Sexual Interactions

The rewarding properties of mountings, intromissions, and ejaculations can be evaluated by conditioning procedures. In fact, the process of conditioning in appetitive behavior usually requires several sessions before there are visible effects. Ultrasound vocalizations in the 50-kHz band seem to be the most robust parameter reflecting positive emotional states (Brudzynski, 2007). In this line, high numbers of PVs convey the rewarding value of previous sexual contacts (Bialy et al., 2000). Additionally, conditioning during a second-order procedure (Everitt et al., 1989), conditioned place preference procedure (Camacho et al., 2009; Tenk et al., 2009), and instrumental conditioned reflexes during copulation (Beck, 1971; Beck et al., 2002) are useful in the evaluation of the rewarding value of subsequent events during sexual interactions. In addition, postejaculatory vocalizations—the most of time, extremely flat long-lasting vocalizations in the 22-kHz frequency band—probably reflect abrupt decreases in sexual arousal and a relaxation state following ejaculation. Thus, in this sense, these 22-kHz vocalizations can be used as an additional measure of reduction in sexual arousal and motivation related to the preceding ejaculation (Bialy et al., 2016). Postejaculatory vocalizations usually co-occur with a male’s inactivity or grooming. In addition, we found that males vocalize for significantly longer when a female is present in the copulatory chamber after ejaculation (Sachs and Bialy, 2000). Furthermore, such vocalizations are present only in a familiar environment, and cues that increase anxiety level (odor cues from unfamiliar males) significantly reduce such postejaculatory vocalization (Bialy et al., 2016). Moreover, the postejaculatory vocalizations are distinct from shorter low-frequency vocalizations that are produced by a male rat expressing a sexually related frustration state (Bialy et al., 2019).

## Rat Sexual Behavior and Human Diseases Associated with Sexual Dysfunction

Table 1 summarizes the key components of rat sexual behavior with relevant parameters that describe specific aspects of the behavior (first column). These parameters may be used to quantify disturbances of sexual motivation and performance (second column) that are observed in various rodent models of human diseases (third column). Moreover, additional parameters and tests can be used to further delineate and confirm the underlying causes of abnormal sexual behavior (fourth column), for example, level of anxiety or anhedonia. Furthermore, analysis of these parameters may be useful for evaluation of the efficacy of therapeutic interventions in preclinical investigations.

**Table 1:** Particular components of male sexual behavior, parameters that describe them and their relation to abnormal sexual motivation, arousal and performance due to psychiatric, cardiovascular, neurologic, endocrine and metabolic health disorder, and additional tests to confirm causes of changes in parameters in the rat models.

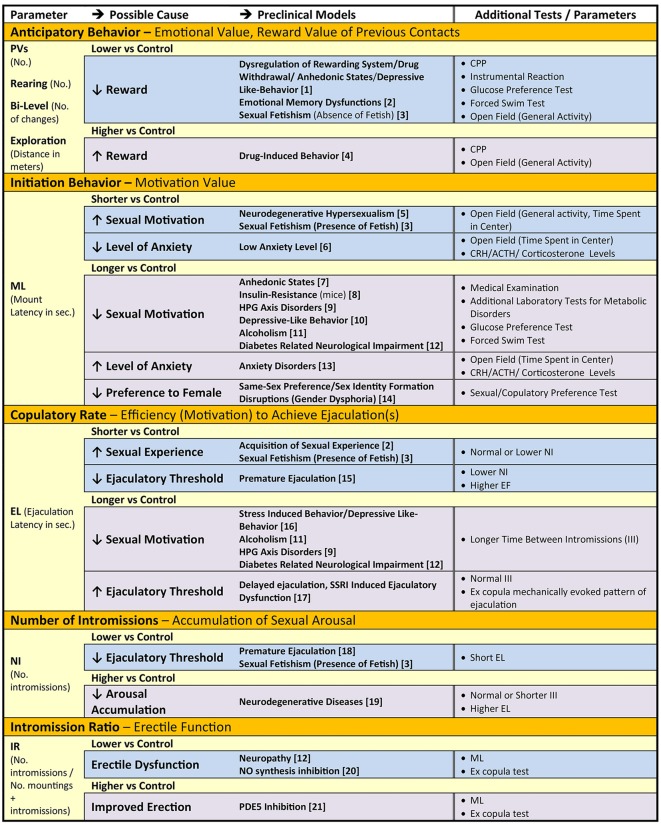

*Since sexual behavior depends on specific strain of rats, laboratory environment, nutrition and housing (Hansen et al., 1978; Bialy et al., 2014; Molenda-Figueira et al., 2017; Sanna et al., 2017), changes in specific parameters describing male sexual behavior should be evaluated against values obtained in control groups in a given experimental paradigm. Abbreviations: ACTH, adrenocorticotropic hormone; CRH, corticotropin-releasing hormone; EF, ejaculation frequency; HPG, hypothalamic-pituitary-gonadal axis; III, inter-intromission interval; PDE5, phosphodiesterase 5; PVs, precontact vocalizations in the 50-kHz band; SSRI, selective serotonin reuptake inhibitor. References in the table: [1] (Pfaus and Phillips, 1991; Van Furth et al., 1994; Barr et al., 1999); [2] (Bialy et al., 2000); [3] (Pfaus et al., 2013); [4] (Fiorino and Phillips, 1999); [5] (Novati et al., 2018); [6] (Barrot et al., 2005; Miwa et al., 2011); [7] (Pfaus and Phillips, 1991); [8] (Faulkner et al., 2015); [9] (Babaei-Balderlou and Khazali, 2016); [10] (Bialy et al., 2014); [11] (Sadeghzadeh et al., 2018); [12] (McVary et al., 1997); [13] (Hawley et al., 2013; Sanna et al., 2014); [14] (Ramírez-Rodríguez et al., 2017; Hernández and Fernández-Guasti, 2018); [15] (Coolen et al., 1997; Pattij et al., 2005a; Clément et al., 2007; Kang et al., 2013; Olayo-Lortia et al., 2014); [16] (Grønli et al., 2005); [17] (de Jong et al., 2005; Hueletl-Soto et al., 2012); [18] (Coolen et al., 1997; Beck and Bialy, 2000); [19] (Harris and Sachs, 1975; Valcourt and Sachs, 1979; Novati et al., 2018); [20] (Hull et al., 1994; Bialy et al., 1996); [21] (Ferraz et al., 2016)*.

The translational application of animal models should be exploited cautiously, as not all aspects of sexual behavior and health disorders are identical in rats and humans (Le Moëne and Ågmo, 2019). Additionally, rodent models usually comprise only selected aspects of the complex pathogenesis of neurological, cardiovascular, and metabolic diseases in humans (Zaragoza et al., 2011; Dawson et al., 2018; Lutz, 2018). Even though the sexual behavior of a male rat is not identical to that seen in humans, neurotransmitters, brain structures, and neuronal networks and the motivational, and consummatory aspects of the sexual behavior seem to be fundamentally similar (Larsson and Ahlenius, 1999; Pattij et al., 2005b; Chan et al., 2008; Georgiadis et al., 2012). Since the sexual behavior of a male rat is well defined in terms behavioral, anatomical, and neurochemical characteristics, investigation of sexual behavior in various rodent models of human diseases provides a translational framework for better recognition of the underlying mechanisms of the sexual dysfunction seen in numerous human health disorders and their potential treatment.

## Author Contributions

MB conceived the study, analyzed the literature, prepared the figure, wrote and revised the manuscript, and secured funding. WB-R analyzed the literature, prepared the table, and wrote the manuscript. JP analyzed the literature, reviewed the manuscript, and secured funding. TZ analyzed the literature, prepared the table, and wrote and revised the manuscript.

## Conflict of Interest

The authors declare that the research was conducted in the absence of any commercial or financial srelationships that could be construed as a potential conflict of interest.
